# Damage mechanisms in defected natural fibers

**DOI:** 10.1038/s41598-017-14514-6

**Published:** 2017-10-25

**Authors:** Johnny Beaugrand, Sofiane Guessasma, Jean-Eudes Maigret

**Affiliations:** 1 INRA, UMR614 FARE, Fractionnement des AgroRessources et Environnement, 2 esplanade Roland Garros, F-51100 Reims, France; 2 INRA, Research Unit BIA UR1268, Rue Geraudiere, F-44316 Nantes, France

## Abstract

A novel experimental setup is presented to reveal damage mechanisms in bast fibers. 3D imaging at submicronic scale based on X-ray micro-tomography is combined with *in-situ* tensile experiments of both elementary fibers and bundles. The results reveal that the relevant scale that drives failure of hemp lignocellulosic fibers is submicronic. *In-situ* tensile experiments assisted by X-ray micro-tomography shows complex damage mechanisms involving the constitutive sub-layer structure, fiber extraction defects like kink bands, and the tubular porosity of the natural fiber.

## Introduction

Research effort on natural fibers may appear as giving into fads because, for some people, the green label matters more than the performance. It is true that economic attractiveness of natural fibers goes with the first signs of major crisis, justified mostly by the crude oil prices. For acting scientists, natural fibers are legitimate contrasts to synthetic fibers because natural fibers reflect a world of diversity^1^, and may uphold a commercial value if plotted from the perspective of the new concept of bioeconomy^2–6^. Whether the plant cell wall is named primary (rich in pectin components), or secondary (enriched with semi-crystalline cellulose), it is a subject of major scientific focus^7^. The roadmap drafted by scientists to gain better knowledge about natural fiber organization has real implications in material engineering starting from bioinspired design to composite manufacturing^2,6^.

**Figure 1:**
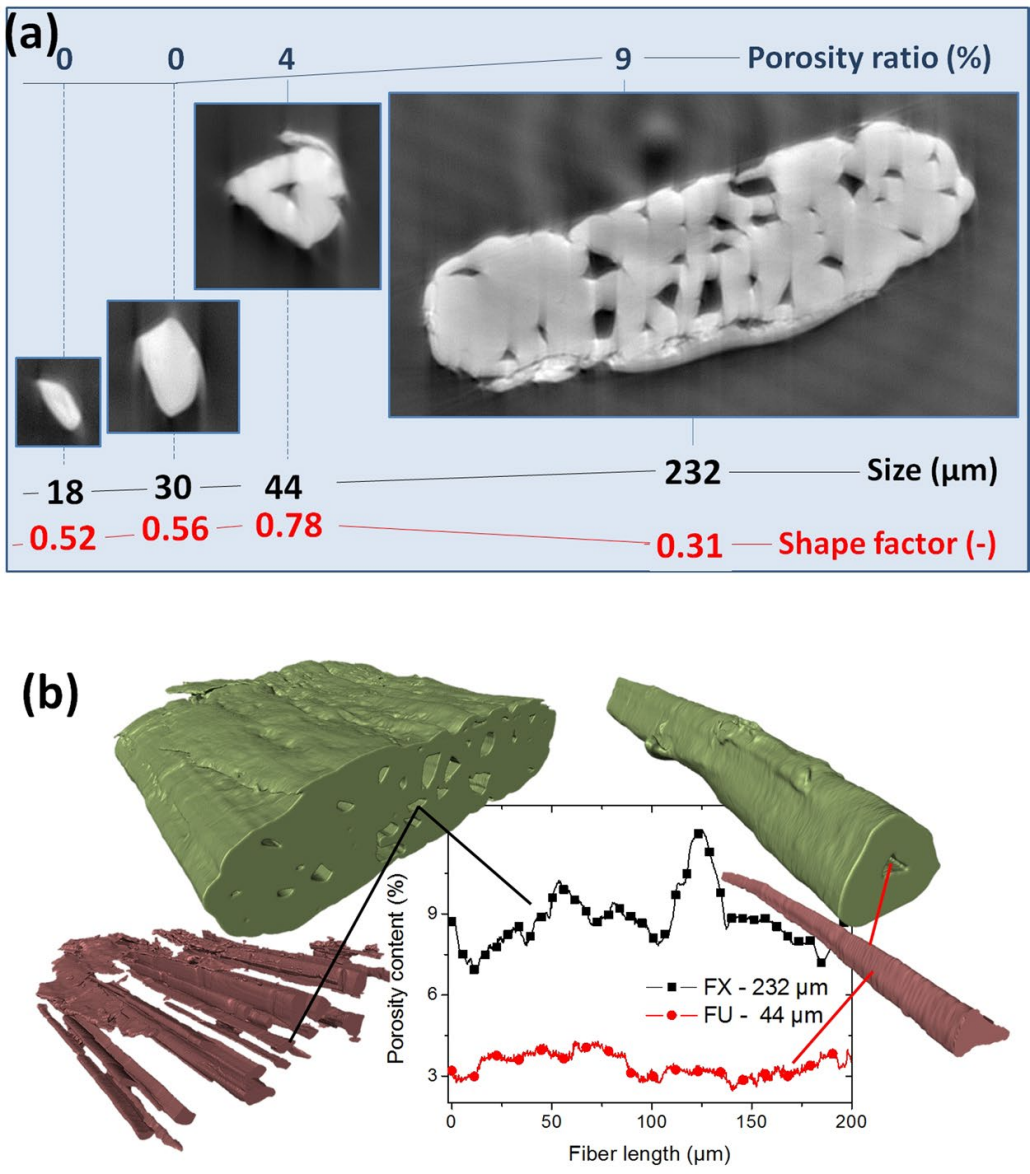
(**a**) Cross-section views of tomograms revealing hemp fiber internal arrangement. Lumen appears at a characteristic length of 40 µm together with surface flaws. (**b**) Porosity profiles along the fiber length for one bundle (on the left) and one elementary fiber (on the right) of contrasted dimensions.

The structure of the natural fiber is far from being untidy. A hierarchical organization spreading from the nano-to the millimeteric scale is a proof of its deliberate complexity^8^. Hemp bast fiber elements exhibit a thin primary cell wall (outward) that connect individual fibers together in bundles, and a thick secondary cell wall (onward) organized in wrapped sub-layers^9^. An empty space, the lumen, is more or less present in the fiber. This space is also referred to as the fiber porosity. Some authors report porosity content between 7 to 12% in every individual fiber based on SEM observations^10–12^. The origin of ‘defected fibers’ in our title comes from two facts reported by Hughes^13^. The first fact relies on intrinsic ultrastructural organization of the fiber, which is shaped by external stresses like the wind, and marked by the great disparity in fiber dimensions and lumen volumes^12^. For those more familiar with uniformity of synthetic fibers, this structure may appear as a genetic condition. They do score a point because, at the sub-micronic level, interfaces are weakness points well known in pulp and paper industry. At the nano scale, the predominant polymer is cellulose. Usually semi-crystalline, cellulose is organized as a microfibrillar oriented structure having the role of cell wall reinforcement, like carbon fibers in a polymer^14^. Microfibrils are embedded in a matrix of amorphous polymers. Most of these polymers are noncellulosic polysaccharides (hemicelluloses and pectin substances). In addition, polyphenol (lignins) is heterogeneously located within the microfibril^15^ but distributed following a logic of gradient across the cell wall sublayer from the fiber cell lumen to the outmost part, the middle lamella^16^. In the secondary thick walls type, cellulose microfibrils have a preferential alignment according to the normal axis of the fiber, identified by the Microfibril Angle (MFA). The MFA varies according to the botanical origins of the fibers and also varies depending on the walls sub layers (S_1_, S_2_, S_3_, etc., up to nine reported in bamboo), with reported drastic MFA transition between sub-layers. The S_2_ is the one where the MFA is the smallest, meaning that the microfibrils of cellulose are oriented almost perpendicular to the normal axis of the fiber. The correlation between MFA and fiber toughness is out of controversy and a good review can be found in the report of Burgert and Fratzl^17^. From one end, the local variation of the cellulose MFA has some credit to explain toughness dispersion of natural fibers. In fact, MFA variability occurs during the growth of the cell wall due to orientation changes of the cellulose-synthesizing machinery^7^. But from the other end, MFA variability does not hold most of the explanation as further detailed here. Figure 1a shows the hierarchical structure of typical bast fiber from hemp plant investigated using X-ray micro-tomography. The fine resolution of imaging (voxel size of 280 nm) is selected to reveal defects of submicronic nature of the fiber along a length scale of 0.5 mm. However, reliable statistics about the length of the elementary fibers cannot be easily achieved as these tend to extend over the field of view. Indeed, secondary fibers are known to be few millimeters in length whereas the primary fibers exceed the centimeter scale^18^. Structural complexity increases from the elementary fiber (on the left side) to the bundle (on the right side). The size limit between an elementary fiber and a bundle can be reasonably discriminated by the presence of more than one lumen. In Fig. 1a, this limit is close to 40 µm. The degree of complexity is fairly guided by the largest dimension of the fiber cross-section, D or major axis length. It reflects change in surface topography from smooth undamaged contour (D < 40 µm) to jagged profile with multiple surface flaws (D > 100 µm). Fiber shape evolves from elongated (D < 20 µm; Shape factor 0.52) to rounded (D < 50 µm; shape factor 0.78) and back to elongated (D > 100 µm; shape factor 0.31) as a result of association of multiple elementary fibers. Mesoscopic porosity appears at a characteristic length of 40 µm. It reaches its maximum value of 8.8% ± 1.1% when several elementary fibers are jointed. Figure 1a demonstrates that there is a correlation between the fiber cross-section area and the void content starting from a characteristic dimension of 40 µm. Examination of the porosity content profiles along the fiber length shows tubular porous network (Fig. 1b). The internal void has submicronic lateral dimension but extends over the entire fiber length. This porosity is thought to play a central role in driving fiber failure by mechanisms of decohesion. In the following, we show that this vision is shorter than the reality revealed by *in-situ* measurements. The new *in-situ* setup proposed in this study captures the dynamics of bast fiber rupture at a fine scale allowing for 3D submicoronic details as small as 280 nm to be observed.

## The ruptured fiber beyond surface flaws

**Figure 2:**
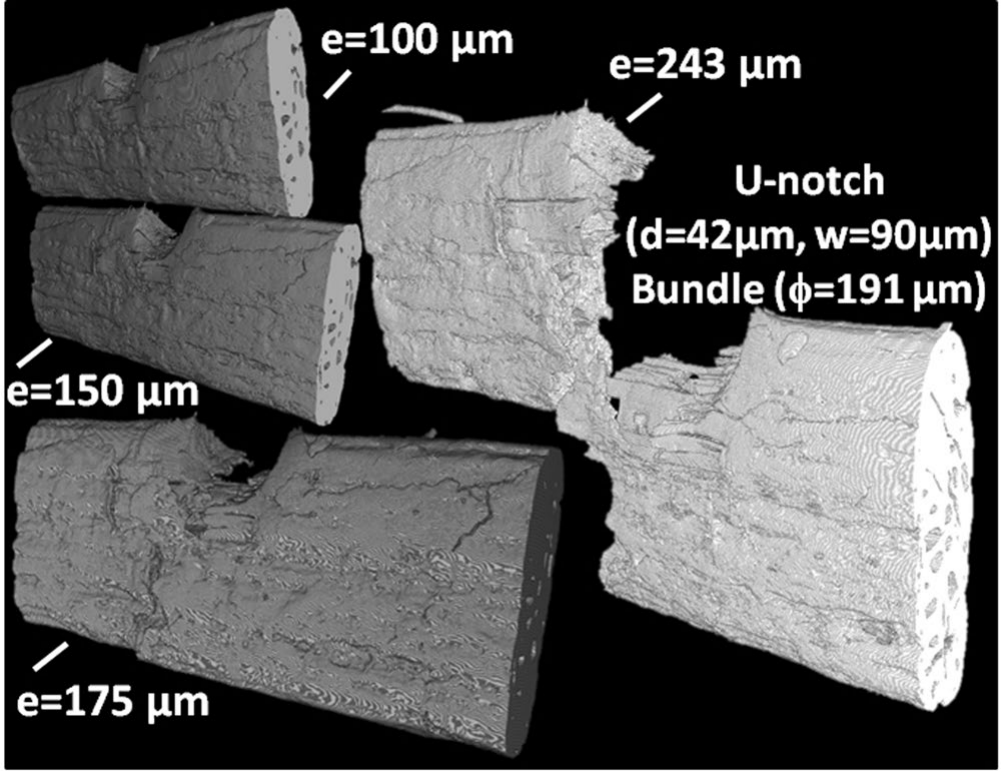
Rupture under tension of bast fiber bundle. e: displacement, d: notch width, w- notch height, ϕ: largest cross-section dimension.

**Figure 3:**
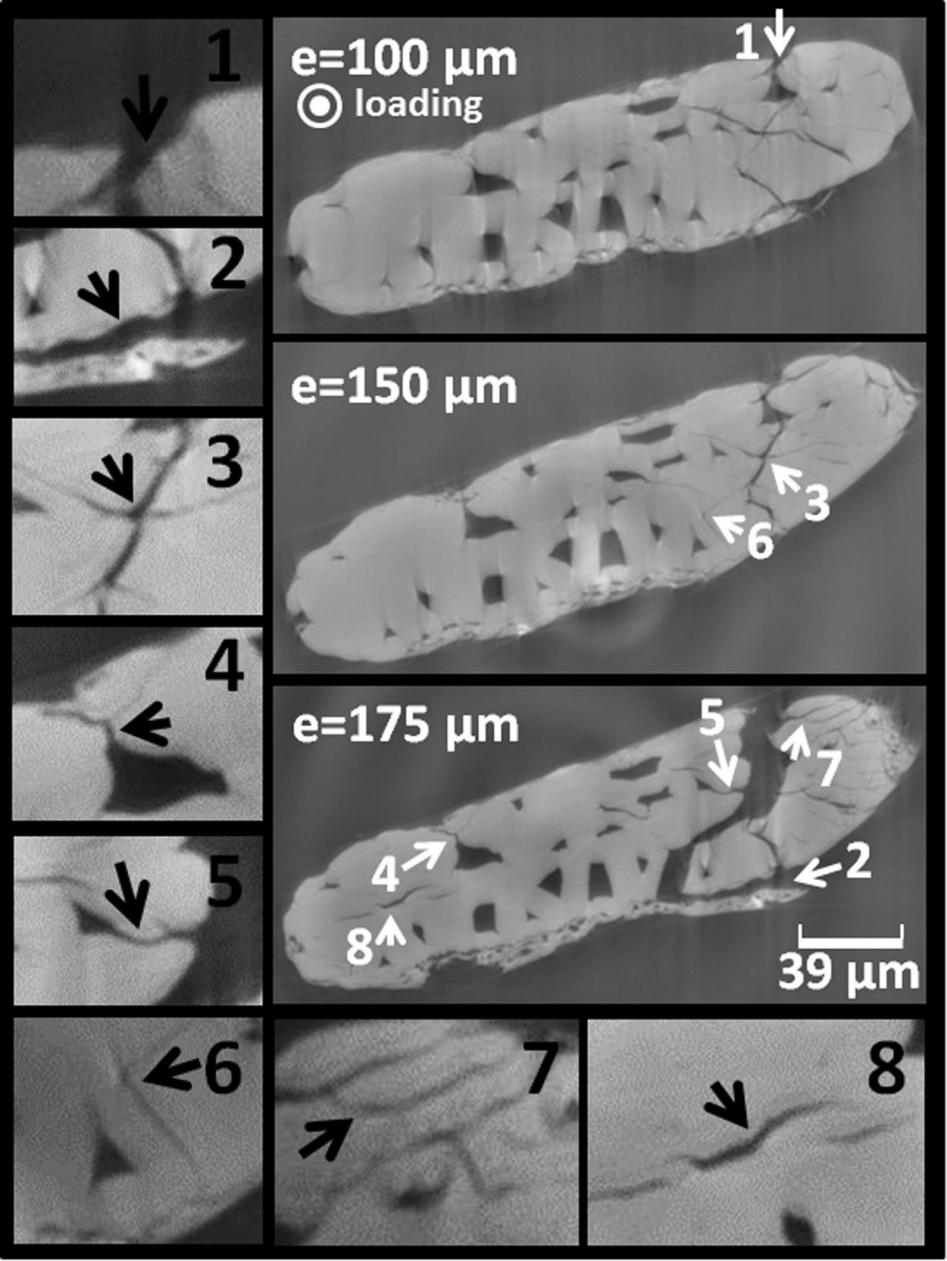
Damage mechanisms in bast fiber bundle (e: magnitude of tensile loading): 1: µ-crack departure from surface flaw (crack depth 3 µm), 2: surface peeling, 3: crack branching, 4: lumen crack departure, 5: intra-lumen damage, 6: inter-lumen damage, 7: inter-fiber cracking and branching, 8: interfacial cracking.

The second fact related to defected natural fibers is originated from mechanical extraction of fibers and bundles. It involves the first defibrization process (decortication) and the cascade transformation (partial peeling of external sublayer). Indeed, this action results in pits, partial delamination of constitutive sub wall layers, and compression or kink bands inside individual or bundles fibers. When it comes to facts, both structural and machining considerations contribute to the observed negative correlation between stiffness and fiber geometry^19^. Defects do not rule out the influence of MFA widely reported^20–23^ and cellulose content^23^. With these arguments in hand, failure properties of natural fibers are still not clearly captured because of lack of defect representation in conceptual models^24,25^. Studies materializing cell wall/lumen ratio effect^1,26^ report a first view of the defect influence on fiber performance. Yet, surface flaws are not fully introduced and defects at the ultrastructural level are far from being championed. Figure 2 shows the complex rupture pattern associated with tensile loading of notched natural bundle determined using X-ray micro-tomography. Notching provides necessary curvature for stress localization to drive failure over other surface defects naturally present in the fiber. Loaded fiber exhibits crack deviation from the opening mode and the tearing type failure is evident^25^. The failure model of the fiber combines several deformation mechanisms that are apprehended from *in-situ* observation of the deformed ultra-structure. Figure 3 shows the collection of mechanisms identified from the cross-section views at successive loading points. The first mechanism connects surface flaws to lumen and contributes to transverse cracking (mechanism #1). Surface peeling (mechanism #2) appears as a result of shearing that take place on the fiber surface. This is a form of sub-layer delamination that is localized at the outer part of the bundle. Several mechanisms involve the weak imperfect interfaces between elementary fibers. Among these, we mention the crack branching (mechanisms #3 and #7) and crack initiation or deviation at the interface of fiber elements (mechanisms #6, #8). At least two other mechanisms are associated to lumen including crack departure from these locations acting as stress concentrators (mechanism #4) or crack annihilation (mechanism #5).

**Figure 4:**
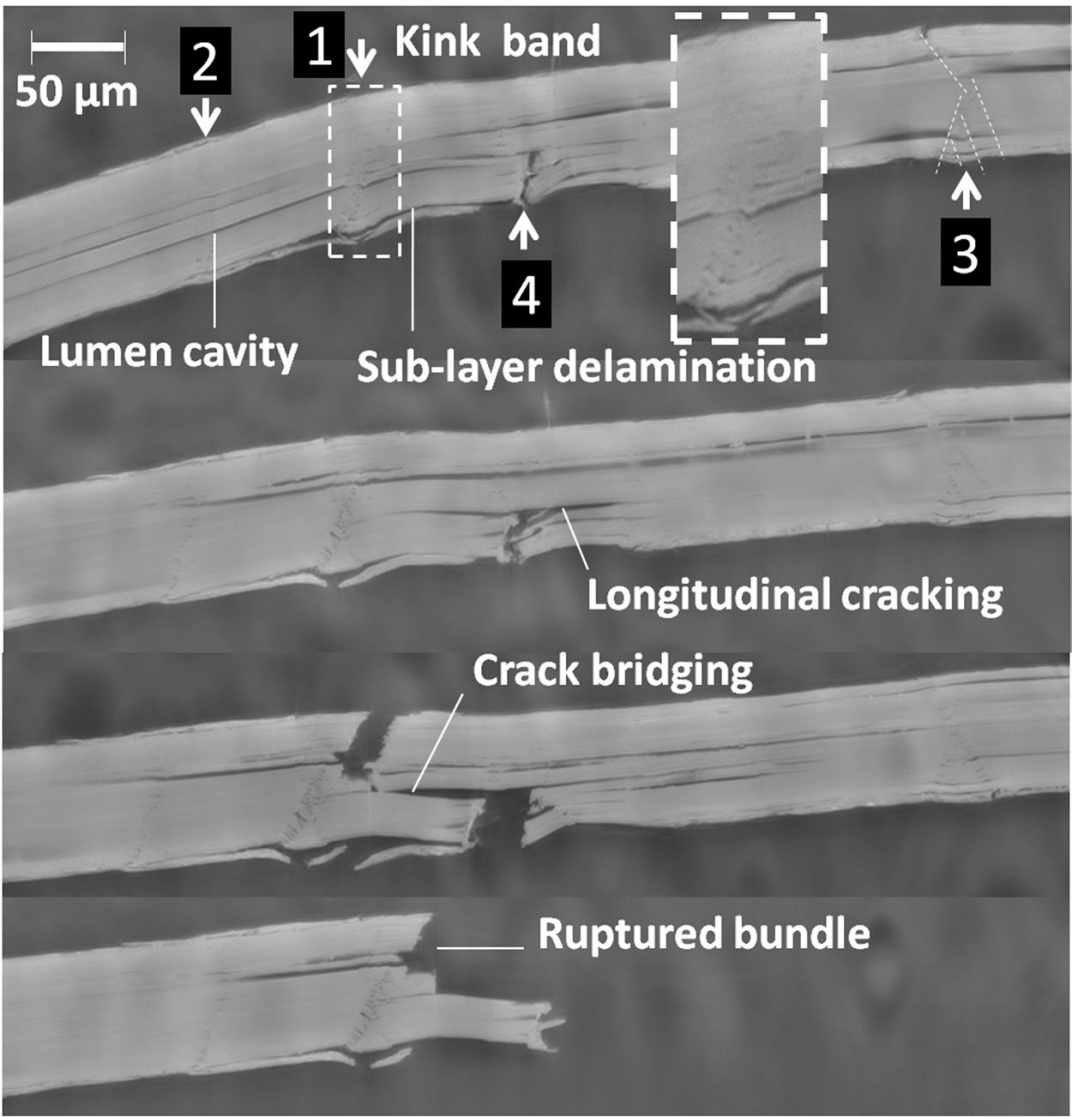
Longitudinal cross-sections acquired at different load levels showing the role of kink bands and the extent of crack branching along the bundle length.

The in-plane extent of revealed damage questions the relationship with load direction. Figure 4 illustrates the role of second type of defects induced during fiber extraction, namely kink bands and sub-layer delamination. At the submicronic scale, characteristic pre-damage in the porous structure along inclined directions is symptomatic of the presence of kink bands (label 3 on the top longitudinal view in Fig. 4). The density of kink bands is significant along the fiber length and exhibits a large dispersion (4.45–15.80 mm^−1^). The longitudinal views at different load levels reveal that kink bands can be preferential sites for crack departure (label #4), active (label # 1) or passive (label #3) pre-damages depending on the amount of transferred load. The subsequent crack growth follows an inclined path in contrast to a mode I propagation, which tends to produce a trajectory perpendicular to the loading direction. This mixed mode crack opening involves in-plane shearing and is controlled by the characteristic pre-damage genuine to the kink band. The lumen space deviates the transverse cracking initiated from the kink band to longitudinal one making it possible to multiple transverse cracks to connect (second view from the bottom) and lead to failure (first view from the bottom). This mechanism has been identified from optical measurements as longitudinal splitting in flax fibers^27^. This is the main idea of crack bridging illustrated in Fig. 4, as a possible rupture scenario in presence of a large density of kink bands. This scenario also involves a minor role of sub-layer delamination and a central role of the lumen cavity. Significant delaying of transverse cracking can be achieved depending on how lengthy is the lumen cavity (from 149 ± 61 µm to lengths exceeding the field of view of 573 µm). If the availability of this lumen space is rationally related to the length of the elementary fiber, this provides a clear picture of the role of elementary fiber on bundle tensile behavior.

**Figure 5:**
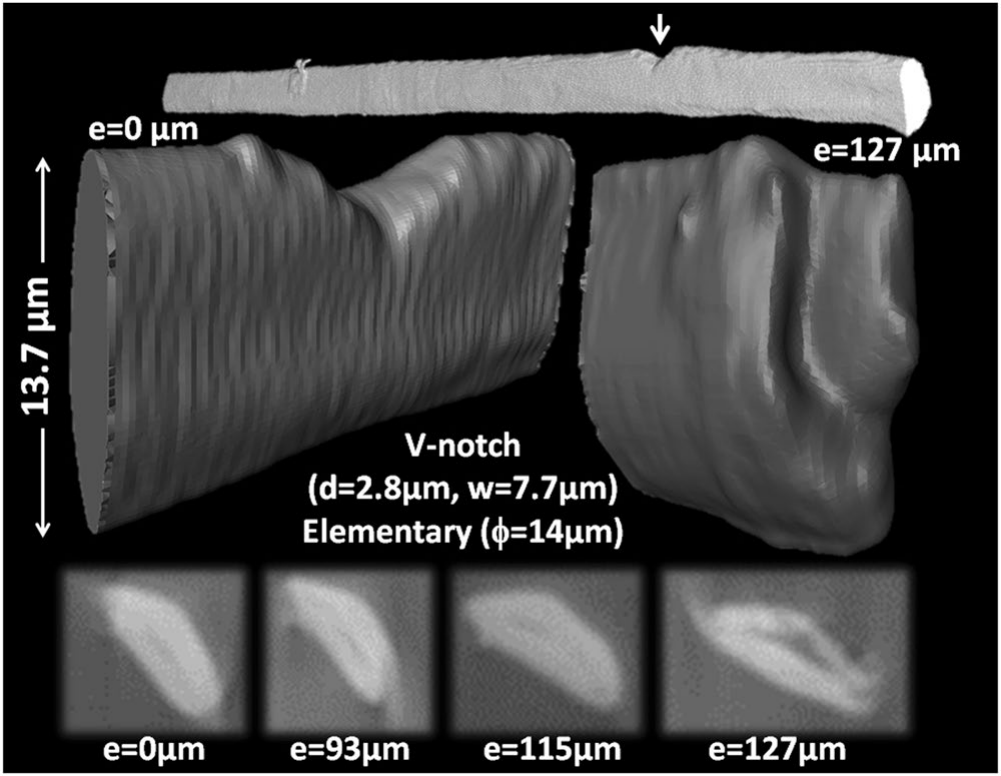
Rupture under tension of elementary bast fiber. e: displacement, d: notch width, w- notch height, ϕ: largest cross-section dimension.

A contrasted example to the multiple damage mechanisms is shown in Fig. 5 for an elementary fiber exhibiting a lower complexity. Crack deviation indicates the role of structural heterogeneity, which can be related to either traces of lumen or internal damage responsible for the observed rupture profile.

## Discussion of the size effects

Dispersion of tensile properties of bast fibers can be approached using the weak link theory, which predicts the failure based on the existence of defects. When Weibull analysis is used, for instance, to correlate this dispersion to the size of elementary fiber, it is simply a matter of measuring the effect of kink bands^28^. Former results on flax fibers^29^ and the results shown in Fig. 4 support the idea of an inter-defect distance as a key quantity to be related to the strength variability in natural fibers. A previous study performed on a large number of fibers and involving high speed camera recording showed, in addition, a ranking of the damage mechanisms according to the lateral dimension of the bast fibers^30^. From Fig. 1, this ranking appears related to the correlation between the void content and the volume of the fiber, or more exactly the cross-section area starting from a characteristic dimension of 40 µm. From Fig. 4, this ranking is also correlated to the density of process-induced defects along the length of the fiber. The combination of scale dependent tubular porosity and randomly distributed defects is the explanation of the variability in mechanical performance of bast fibers.

The level complexity of the damage mechanisms is undoubtedly increased when natural fibers are used as fillers or reinforcements in composites. The load transfer between the matrix and the natural fiber combines the failure mechanisms inherit to the fibers with the interfacial mechanisms that depend on other considerations such as quality of the fiber – matrix adhesion, fiber content and geometrical arrangement of phases in the composite^29,31,32^.

## Conclusions

The proposed *in-situ* imaging setup based on X-ray micro-tomography demonstrates the relevance of submicronic observations to capture the rupture behavior in bast fibers. Leading damage mechanisms in bast fibers are scale dependent. Transverse cracking predominates for elementary fibers. However, the submicronic complexity and the tubular nature of the porous structure defend the idea of a hierarchical rupture for bundles where multiple mechanisms are simultaneously activated. It is well admitted that delaying the bast fiber failure is a matter of lowering the weight of surface flaws. Surface treatment of bast fibers, although privileged in various engineering situations, comes at a certain cost (adding a step in the process, losing some of the green value because of the chemicals involved, etc.). Predicting the bast fiber mechanical performance is still challenging because of the combined role of a scale dependent porosity and a process-induced pre-damage.

From the composite engineering perspective, the debate is not whether natural fibers still attract in spite of their failure complexity, it is more about how to use this complexity in the engineering design. A way to explore for preventing premature rupture is to involve the genetics of the plant by enhancing the ability of the fiber to deviate cracking longitudinally if the fiber is meant to work in tension. With the same density of surface flaws, superior performance can be achieved if the submicronic porous structure is controlled.

## Controlling damage growth in bast fibers

Due to the significant action of surface flaws in driving unpredictable failure of hemp fibers^30^, guidance of damage is forced using notched specimens at a fine scale.

## Material

Individual fibers and bundles (diameter: 14–191 µm) are provided by Fibres Recherche Développement® (Troyes, France) and were industrially extracted from hemp stems (Cannabis Sativa L, variety Fedora 17) cultivated in Champagne Ardenne, France, and harvested in September 2012 (La Chanvrière company). The industrial batch contains up to 90% of primary fibers against 10% of secondary fibers. Plants were harvested before full seed maturity and a dew retting was done in the field. Fibers elements were stored in controlled environment and maintain an overall moisture content of 7–8%.

## Methods

V- and U- Notches (depth: 3–68 µm, width: 2–90 µm) in natural fibers are realized using laser micro-dissector (PALM® MB IV, ZEISS) mounted on a ZEISS AXIO OBSERVER Z1 (Carl Zeiss, Oberkochen, Germany) inverted microscope. The pulsed laser (wavelength of 355 nm) is operated at a frequency of 100 Hz and beam energy of 90 µJ.

*In-situ* observation of damage growth in hemp fibers under tension is based on X-ray microtomography (beam line ID19, ESRF, Grenoble, France) with voxel size of 280 nm, resolution of 8.6 × 10^9^ voxels, beam energy of 19 keV and 1400 projections for each tomogram. The field of view represents a typical volume of 573 µm^3^. Radiographic images are collected by a Frelon camera (2048 × 2048 pixels) developed at ESRF coupled to GGG scintillator (thickness 10 µm) and an optical × 20/NA 0.45 objective from Olympus. The tensile machine is developed by 3SR laboratory (Grenoble, France). Tension is performed using a tensile bench (load cell of 50 N, load rate of 1.25 µm/second) on specimens of 20 mm in length with 4 load steps up to failure.

Tomograms are built using filter-back projection method and processed using public domain ImageJ software. Processing includes thresholding, wrapping, filtering, and granulometry analysis. Fiber shape factor is derived from the fitted ellipse of the fiber cross-section as:

$$shape\,factor={minor}\,\mathrm{axis}{/}\mathrm{major}\,{axis}{.}$$

### Data availability

X-ray micro-tomography data are available from the authors upon request.
